# Supplementary material for: MicroRNA Profiling in Oesophageal Adenocarcinoma Cell Lines and Patient Serum Samples Reveals a Role for miR-451a in Radiation Resistance
Source: Int J Mol Sci. 2020 Nov 24;21(23):8898. doi: 10.3390/ijms21238898 (PMC7727862; doi:10.3390/ijms21238898)
Supplement: Supplementary file 1 [file ijms-21-08898-s001.zip › supply/Supplementary file 1_FB_GM_DJH.docx]

Supplementary File 1.


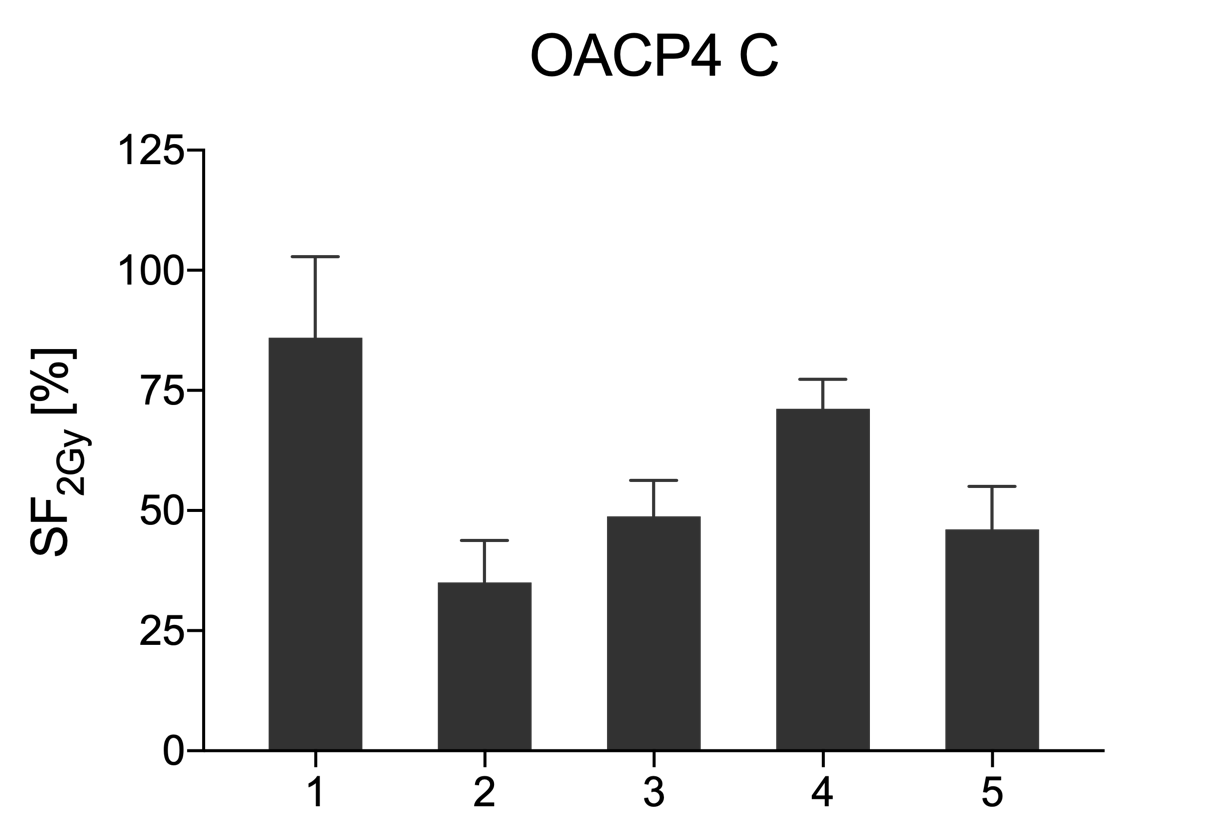


**Supplementary Figure S1.** Intrinsic radiation resistance of cell line OACP4 C, in n=5 independent experiments. Survival Fraction + SD of OAC cell lines measured via clonogenic assay after irradiation with 2 Gy, normalised to negative controls.

**Supplementary Figure S2.** JHEso-Ad1 cells were an outlier in Spearman non-parametric correlations between treatment survival fractions and average centralised miRNA expression levels, for miRNAs that had a correlation p-value < 0.05. **A**. Cisplatin. **B**. F-FU. **C**. Radiation. **Green circles**: JHEso-Ad1 cells. **Red circles**: other cell lines.

**Supplementary Table S1.** Predicted molecular pathways of the mRNA targets of miRNAs that were uniquely associated with radiation response.

| Pathway Name | Pathway uploaded gene count | Genes in InnateDB for this entity | Pathway p-value | Pathway p-value (corrected) |
| --- | --- | --- | --- | --- |
| Intrinsic Pathway for Apoptosis | 17 | 38 | 3.37 x 10^-10^ | 4.83 x 10^-7^ |
| Apoptosis | 27 | 155 | 2.40 x 10^-5^ | 0.001 |
| p53 signalling pathway | 16 | 68 | 2.69 x 10^-5^ | 0.001 |
| Plasma membrane oestrogen receptor signalling | 9 | 24 | 3.05 x 10^-5^ | 0.001 |
| alpha-linolenic (omega3) and linoleic (omega6) acid metabolism | 4 | 10 | 0.004 | 0.038 |
| alpha-linolenic acid (ALA) metabolism | 4 | 10 | 0.004 | 0.038 |

**Supplementary Table S2.** Predicted molecular pathways of the mRNA targets of miRNAs that were uniquely associated with cisplatin response.

| Pathway Name | Pathway uploaded gene count | Genes in InnateDB for this entity | Pathway p-value | Pathway p-value (corrected) |
| --- | --- | --- | --- | --- |
| p53 signalling pathway | 30 | 68 | 3.35 x 10^-9^ | 1.19 x10^-7^ |
| Plasma membrane oestrogen receptor signalling | 11 | 24 | 2.29 x 10^-4^ | 0.002 |
| Pten dependent cell cycle arrest and apoptosis | 8 | 15 | 4.72 x 10^-4^ | 0.003 |
| Caspase Cascade in Apoptosis | 17 | 53 | 9.03 x 10^-4^ | 0.005 |
| Fatty acid, triacylglycerol, and ketone body metabolism | 41 | 176 | 0.001 | 0.006 |
| Intrinsic Pathway for Apoptosis | 13 | 38 | 0.002 | 0.009 |
| PI Metabolism | 15 | 51 | 0.005 | 0.019 |
| Apoptosis | 22 | 88 | 0.006 | 0.025 |

**Supplementary Table S3.** Predicted molecular pathways of the mRNA targets of miRNAs that were uniquely associated with increased 5-FU response.

| Pathway Name | Pathway uploaded gene count | Genes in InnateDB for this entity | Pathway p-value | Pathway p-value (corrected) |
| --- | --- | --- | --- | --- |
| p53 signalling pathway | 21 | 68 | 1.46 x 10^-7^ | 1.80 x 10^-5^ |
| Caspase Cascade in Apoptosis | 13 | 53 | 4.60 x 10^-4^ | 0.007 |
| Pten dependent cell cycle arrest and apoptosis | 6 | 15 | 0.001 | 0.013 |
| Apoptosis | 16 | 88 | 0.003 | 0.027 |

**Supplementary Table S4.** Predicted molecular pathways of the mRNA targets of miRNAs that were uniquely associated with decreased 5-FU response.

| Pathway Name | Pathway uploaded gene count | Genes in InnateDB for this entity | Pathway p-value | Pathway p-value (corrected) |
| --- | --- | --- | --- | --- |
| p53 signalling pathway | 12 | 68 | 1.35 x 10^-5^ | 3.56 x 10^-4^ |
| Intrinsic Pathway for Apoptosis | 9 | 38 | 1.38 x 10^-5^ | 3.57 x 10^-4^ |
| Oestrogen responsive protein efp controls cell cycle and breast tumours growth | 5 | 15 | 2.14 x 10^-4^ | 0.003 |
| Plasma membrane oestrogen receptor signalling | 6 | 24 | 2.86 x 10^-4^ | 0.003 |

**Supplementary Table S5.** Predicted molecular pathways of the mRNA targets of miRNAs that were associated with both the radiation and cisplatin response.

| Pathway Name | Pathway uploaded gene count | Genes in InnateDB for this entity | Pathway p-value | Pathway p-value (corrected) |
| --- | --- | --- | --- | --- |
| Ribosome | 71 | 137 | 6.45 x 10^-11^ | 2.93 x 10^-9^ |
| Apoptosis | 70 | 155 | 1.40 x 10^-7^ | 3.47 x 10^-6^ |
| Intrinsic Pathway for Apoptosis | 21 | 38 | 1.11 x 10^-4^ | 8.46 x 10^-4^ |
| Fatty acid, triacylglycerol, and ketone body metabolism | 66 | 176 | 4.22 x 10^-4^ | 0.003 |
| Clathrin derived vesicle budding | 27 | 60 | 0.001 | 0.006 |
| trans-Golgi Network Vesicle Budding | 27 | 60 | 0.001 | 0.006 |
| Regulation of lipid metabolism by Peroxisome proliferator-activated receptor alpha (PPARalpha) | 42 | 109 | 0.002 | 0.012 |
| Regulation of Apoptosis | 26 | 60 | 0.002 | 0.012 |
| Golgi Associated Vesicle Biogenesis | 23 | 53 | 0.004 | 0.017 |
| PI Metabolism | 22 | 51 | 0.005 | 0.021 |
| Internal ribosome entry pathway | 10 | 18 | 0.007 | 0.026 |
| Metabolism | 436 | 1535 | 0.009 | 0.031 |
| Glycolysis | 12 | 25 | 0.014 | 0.047 |

**Supplementary Table S6.** Predicted molecular pathways of the mRNA targets of let-7d-5p, which were associated with radiation, cisplatin and 5-FU responses.

| Pathway Name | Pathway uploaded gene count | Genes in InnateDB for this entity | Pathway p-value | Pathway p-value (corrected) |
| --- | --- | --- | --- | --- |
| Intrinsic Pathway for Apoptosis | 17 | 38 | 3.37 x 10^-10^ | 4.83 x 10^-7^ |
| Apoptosis | 27 | 155 | 2.40 x 10^-5^ | 0.001 |
| p53 signalling pathway | 16 | 68 | 2.69 x 10^-5^ | 0.001 |
| Plasma membrane oestrogen receptor signalling | 9 | 24 | 3.05 x 10^-5^ | 0.001 |
| p53 pathway | 10 | 47 | 0.002 | 0.023 |
| alpha-linolenic (omega3) and linoleic (omega6) acid metabolism | 4 | 10 | 0.004 | 0.038 |
| alpha-linolenic acid (ALA) metabolism | 4 | 10 | 0.004 | 0.038 |

**Supplementary Table S7. *TP53* mutations in oesophageal adenocarcinoma cell lines, and their consequences.** c., CDNA sequence; p., protein (amino acid) sequence; *, nonsense (noncoding) substitution; ins, nucleotide insertion; fs, frameshift; SIFT, Sorting Intolerant From Tolerant (program that predicts the effects of amino acid substitutions upon protein function) [1]; COSMIC, Catalogue of Somatic Mutations in Cancer [2]; IARC, International Agency for Research on Cancer *TP53* mutation database [3]; na, not available.

| **Cell line.** | ***TP53* DNA Mutation** | **p53 Protein Mutation** | **p53 Domain** | **Phenotype** | **Transcriptional Activity Class** | **SIFT Prediction** | **Reference** |
| --- | --- | --- | --- | --- | --- | --- | --- |
| Eso-26 | c.742C>T | p.R248W | DNA binding domain | Missense substitution | Non-functional | Deleterious | COSMIC, IARC |
| Eso-51 | c.524G>A | p.R175H | DNA binding domain | Missense substitution | Non-functional | Deleterious | COSMIC, IARC |
| Flo-1 | c.830G>T | p.C277F | DNA binding domain | Missense substitution | Non-functional | Deleterious | COSMIC, IARC |
| JH-EsoAd1 | c.797G>A | p.G266E | DNA binding domain | Missense substitution | Non-functional | Deleterious | [4], IARC |
| OACP4C | c.574C>T | p.Q192* | DNA binding domain | Nonsense substitution  (? Truncation) | na | na | COSMIC, IARC |
| OE-19 | c.927_928insA | p.N310fs*27 | Homooligomerisation domain | Frameshift insertion |  |  | COSMIC |
|  | c.929_930ins1 | p.? | Homooligomerisation domain | Frameshift insertion | na | na | IARC |
| OE-33 | c.404G>A | p.C135Y | DNA binding domain | Missense substitution | Non-functional | Deleterious | COSMIC, IARC |
| SK-GT-4 | c.298C>T | p.Q100* | DNA binding domain | Nonsense substitution  (? Truncation) | na | na | COSMIC, IARC |
|  | c.524G>A | p.R175H | DNA binding domain | Missense substitution | Non-functional | Deleterious | IARC |

References

1. Sim, N.-L., et al., *SIFT web server: predicting effects of amino acid substitutions on proteins.* Nucleic Acids Research, 2012. **40**(W1): p. W452-W457.

2. Forbes, S.A., et al., *COSMIC: somatic cancer genetics at high-resolution.* Nucleic Acids Res, 2017. **45**(D1): p. D777-D783.

3. Bouaoun, L., et al., *TP53 Variations in Human Cancers: New Lessons from the IARC TP53 Database and Genomics Data.* Hum Mutat, 2016. **37**(9): p. 865-76.

4. Alvarez, H., et al., *Establishment and characterization of a bona fide Barrett esophagus-associated adenocarcinoma cell line.* 2008. **7**(11): p. 1753-5.

**Supplementary Table S8.** House Keeping Gene information for Chemotherapeutic drug treated cell lines.

| **OpenArray ID** | **miRBase v22 sequence** | **miRBase v22 ID** | **miRBase Accession number** |
| --- | --- | --- | --- |
| 002324_hsa-miR-744 | UGCGGGGCUAGGGCUAACAGCA | hsa-miR-744-5p | MIMAT0004945 |
| 000545_hsa-miR-331 | GCCCCUGGGCCUAUCCUAGAA | hsa-miR-331-3p | MIMAT0000760 |
| 000411_hsa-miR-28 | AAGGAGCUCACAGUCUAUUGAG | hsa-miR-28-5p | MIMAT0000085 |
| 002446_hsa-miR-28-3p | CACUAGAUUGUGAGCUCCUGGA | hsa-miR-28-3p | MIMAT0004502 |
| 000390_hsa-miR-15b | UAGCAGCACAUCAUGGUUUACA | hsa-miR-15b-5p | MIMAT0000417 |
| 000497_hsa-miR-197 | UUCACCACCUUCUCCACCCAGC | hsa-miR-197-3p | MIMAT0000227 |
| 002187_hsa-miR-942 | UCUUCUCUGUUUUGGCCAUGUG | hsa-miR-942-5p | MIMAT0004985 |
| 002276_hsa-miR-222 | AGCUACAUCUGGCUACUGGGU | hsa-miR-222-3p | MIMAT0000279 |
| 002283_hsa-let-7d | AGAGGUAGUAGGUUGCAUAGUU | hsa-let-7d-5p | MIMAT0000065 |
| 002349_hsa-miR-574-3p | CACGCUCAUGCACACACCCACA | hsa-miR-574-3p | MIMAT0003239 |
| 000405_hsa-miR-26a | UUCAAGUAAUCCAGGAUAGGCU | hsa-miR-26a-5p | MIMAT0000082 |
| 001097_hsa-miR-146b | UGAGAACUGAAUUCCAUAGGCUG | hsa-miR-146b-5p | MIMAT0002809 |
| 000402_hsa-miR-24 | UGGCUCAGUUCAGCAGGAACAG | hsa-miR-24-3p | MIMAT0000080 |

**Supplementary Table S9.** House Keeping Gene information for Radiation treated cell lines.

| **OpenArray_ID** | **miRBase_v22_sequence** | **miRBase_v22_ID** | **miRBase_Accession number** |
| --- | --- | --- | --- |
| 000411_hsa-miR-28 | AAGGAGCUCACAGUCUAUUGAG | hsa-miR-28-5p | MIMAT0000085 |
| 001097_hsa-miR-146b | UGAGAACUGAAUUCCAUAGGCUG | hsa-miR-146b-5p | MIMAT0002809 |
| 002271_hsa-miR-185 | UGGAGAGAAAGGCAGUUCCUGA | hsa-miR-185-5p | MIMAT0000455 |
| 002324_hsa-miR-744 | UGCGGGGCUAGGGCUAACAGCA | hsa-miR-744-5p | MIMAT0004945 |
| 002187_hsa-miR-942 | UCUUCUCUGUUUUGGCCAUGUG | hsa-miR-942-5p | MIMAT0004985 |
| 000497_hsa-miR-197 | UUCACCACCUUCUCCACCCAGC | hsa-miR-197-3p | MIMAT0000227 |
| 002349_hsa-miR-574-3p | CACGCUCAUGCACACACCCACA | hsa-miR-574-3p | MIMAT0003239 |
| 002276_hsa-miR-222 | AGCUACAUCUGGCUACUGGGU | hsa-miR-222-3p | MIMAT0000279 |
| 000563_hsa-miR-374 | UUAUAAUACAACCUGAUAAGUG | hsa-miR-374a-5p | MIMAT0000727 |
| 000405_hsa-miR-26a | UUCAAGUAAUCCAGGAUAGGCU | hsa-miR-26a-5p | MIMAT0000082 |
| 000545_hsa-miR-331 | GCCCCUGGGCCUAUCCUAGAA | hsa-miR-331-3p | MIMAT0000760 |
| 001020_hsa-miR-365 | UAAUGCCCCUAAAAAUCCUUAU | hsa-miR-365a-3p | MIMAT0000710 |
| 002446_hsa-miR-28-3p | CACUAGAUUGUGAGCUCCUGGA | hsa-miR-28-3p | MIMAT0004502 |
| 000397_hsa-miR-21 | UAGCUUAUCAGACUGAUGUUGA | hsa-miR-21-5p | MIMAT0000076 |

**Supplementary Figure S3.** Comparison of the geometric mean of the House Keeping Gene (HKG) relative levels in cell lines used for chemotherapy drug treatments, with the geometric mean of the HKG levels in cell lines used for radiation sensitivity experiments. Note: OACP4C cells were only used in the chemotherapy drug treatments experiments.

**Supplementary Table S10.** Clinical feature of patients with Oesophageal adenocarcinoma whose neoadjuvant treatment included Radiation and Carboplatin.

|  |  | **TRG 1** |  | **TRG 2** |  | **TRG 3** |  | **TRG 4** |  | **TRG 5** |  |
| --- | --- | --- | --- | --- | --- | --- | --- | --- | --- | --- | --- |
| **Total (n)** |  | **6** |  | **12** |  | **9** |  | **8** |  | **4** |  |
|  |  |  |  |  |  |  |  |  |  |  |  |
| **Age** |  |  |  |  |  |  |  |  |  |  |  |
| Median (years) |  | 64 |  | 66 |  | 60 |  | 67,5 |  | 68,5 |  |
| Range (years) |  | 56 - 72 |  | 64 - 72 |  | 45 - 68 |  | 58 - 82 |  | 50 - 82 |  |
| **Gender** |  |  |  |  |  |  |  |  |  |  |  |
| Male |  | 4 |  | 5 |  | 9 |  | 8 |  | 3 |  |
| Female |  | 1 |  | 0 |  | 0 |  | 0 |  | 1 |  |
| unknown |  | 1 |  | 7 |  | 0 |  | 0 |  | 0 |  |
| **T stage** |  |  |  |  |  |  |  |  |  |  |  |
| cT1 |  | 1 |  | 0 |  | 0 |  | 0 |  | 0 |  |
| cT2 |  | 0 |  | 0 |  | 0 |  | 2 |  | 0 |  |
| cT3 |  | 4 |  | 10 |  | 9 |  | 4 |  | 4 |  |
| cT4 |  | 0 |  | 2 |  | 0 |  | 1 |  | 0 |  |
| cTx |  | 1 |  | 0 |  | 0 |  | 1 |  | 0 |  |
| **N stage** |  |  |  |  |  |  |  |  |  |  |  |
| cN0 |  | 1 |  | 5 |  | 0 |  | 3 |  | 0 |  |
| cN1 |  | 2 |  | 3 |  | 4 |  | 1 |  | 3 |  |
| cN2 |  | 3 |  | 2 |  | 5 |  | 3 |  | 1 |  |
| cN3 |  | 0 |  | 1 |  | 0 |  | 0 |  | 0 |  |
| cNx |  | 0 |  | 1 |  | 0 |  | 1 |  | 0 |  |
| **M stage** |  |  |  |  |  |  |  |  |  |  |  |
| cM0 |  | 6 |  | 12 |  | 9 |  | 8 |  | 4 |  |
