# Supplementary material for: MicroRNA Profiling in Oesophageal Adenocarcinoma Cell Lines and Patient Serum Samples Reveals a Role for miR-451a in Radiation Resistance
Source: Int J Mol Sci. 2020 Nov 24;21(23):8898. doi: 10.3390/ijms21238898 (PMC7727862; doi:10.3390/ijms21238898)
Supplement: Supplementary file 1 [file ijms-21-08898-s001.zip › supply/Supplementary file 7 -- pre-therapy serums from patients treated with 5-FU_FB_GM .docx]

**Supplementary data for miRNAs purified from small extracellular vesicles from pre-treatment serum of patients with oesophageal adenocarcinoma who were subsequently treated with a chemoradiotherapy protocol that included 5-FU.**

Patient information.

For the study of miRNA levels in serum small extracellular vesicles from patients whose chemoradiotherapy protocol included 5-fluorouracil (5-FU), only specimens from patients who met the following entry criteria were included: 1) locally advanced or advanced adenocarcinoma; 2) received treatment with either neoadjuvant, definitive, or palliative chemoradiotherapy (cisplatin, 5-fluorouracil, radiation), 3) had histological assessment of neoadjuvant chemoradiotherapy response based on the American Joint Committee Cancer (AJCC) tumor regression grading system [1] or clinical assessment of chemoradiotherapy response (see below for criteria). Patient details are in “5-FU Supplementary Table 1” below.

Response to neoadjuvant chemoradiotherapy.

Patients that were assessed as having a complete response to chemoradiotherapy were classified as good responders, all other patients were classified as poor responders.

The following criteria were used for assessment:

| **Complete response** |  |
| --- | --- |
| Post-surgery | 100 % eradication of tumour on histopathology |
| No surgery | Complete disappearance of tumour at endoscopy and CT, and no local recurrence within 2 years |
| **Near complete response** |  |
| Post-surgery | Complete disappearance of tumour at endoscopy or on macroscopic inspection of resection specimen, but with scattered infrequent tumour cells on histopathology |
| No surgery | complete disappearance of tumour at endoscopy and CT, but local recurrence within 2 years |
| **Good partial response** |  |
| Post-surgery | persistent tumour at endoscopy or on macroscopic inspection of resection specimen but reduced in size by at least 50% compared to pre-chemo assessment. Easily visible confluent tumour cells on histopathology |
| No surgery | persistent tumour at endoscopy, but reduced in size by at least 50% compared to pre-chemo assessment |
| **Poor partial response** |  |
| Post-surgery | persistent tumour at endoscopy or on macroscopic inspection of resection specimen but reduced in size by less than 50% compared to pre-chemo assessment. Easily visible confluent tumour cells on histopathology. |
| No surgery | persistent tumour at endoscopy, but reduced in size by less than 50% compared to pre-chemo assessment |
| **No response** |  |
| Post-surgery | persistent tumour at endoscopy or on macroscopic inspection of resection specimen, and no reduction in size compared to pre-chemo assessment. Easily visible confluent tumour cells on histopathology |
| No surgery | persistent tumour at endoscopy, and no reduction in size compared to pre-chemo assessment |

Blood Collection, small extracellular vesicle isolation, and RNA purification.

See methods in main manuscript.

TaqMan OpenArray® miRNA Profiling.

To measure the levels of the miRNAs that were significantly associated with the response to 5-FU in the oesophageal cancer cell lines, we extracted RNA from small extracellular vesicles isolated from serum samples of patients who were subsequently treated with chemoradiotherapy protocol that included 5-FU, and measured the relative levels of miRNAs (details in Supplementary File 12) using the Open Array® high throughput qPCR platform as previously described in Chiam *et al.* (2015) [5]. For each sample, 3 μl of RNA was reverse transcribed using pre-defined RT- primers (Megaplex™ Primer Human Pool A and Pool B) and the TaqMan® microRNA Reverse Transcription Kit (Life technologies, #4366596). Pre-amplifications were carried out with Megaplex™ PreAmp Pools and TaqMan PreAmp Master Mix on 7.5 μl complementary DNA (cDNA)/sample for each pool. The pre-amplified products (4 μl per sample) were diluted at the recommended 1:40 dilution with 156 μl of RNase-free ultra pure water before loading onto the 384-well TaqMan OpenArray loading plate. PCR runs were performed using the Biotrove OpenArray NT cycler at Flinders Genomics Facility, Flinders University.

House Keeping Gene Selection.

For normalisation of the miRNAs isolated from serum small extracellular vesicles of patients with oesophageal adenocarcinoma we selected 22 miRNAs as HKGs (5-FU Supplementary Table 3), The selection of Housekeeping Genes (HKG) was performed by applying a modified version of the method of Bianchi *et al*. [6]. HKGs were selected using the following criteria: (i) they were expressed in all samples and at high levels (median Ct < 30); (ii) they were not statistically different in tissue comparisons (Mann Whitney U test, *p* > 0.1); (iii) they were not highly variable (coefficient of variation < 2 x standard deviation) and did not contain outliers (samples with levels not within 5-fold of the mean); and (iv) they were correlated at r > 0.7 with the geometric mean of the house keeping genes.

Differential expression analysis.

The miRNAs were normalised using the geometric mean of the selected HKGs. Welch’s t-tests were used to assess the differential expression of miRNAs between good vs. poor responders who were treated with 5-FU as part of their chemoradiotherapy protocol.

**5-FU Supplementary Table 1.** Clinical features of patients with oesophageal adenocarcinoma whose chemoradiotherapy protocol included 5-FU.

| Age at diagnosis | **Pre-therapy Clinical staging** | **Surgical**  **staging** | **Tumour location** | **Chemoradiotherapy**  **treatments** | **Chemotherapy**  **intention** | **response classification** | **resected / non-resected** |
| --- | --- | --- | --- | --- | --- | --- | --- |
| 64 | 60mm tumour | T0N0 | GOJ | Cis/5FU + Rtx. | neoadjuvant | Good | resected |
| 61 | 20mm tumour | T1bN0M0 | Distal | Cis/5FU + 44 Gy in 25 Fr | neoadjuvant | Good | resected |
| 58 | T3N0 | T3N2 | GOJ | Cis/5FU + 54 Gy in 37 Fr | neoadjuvant | Good | resected |
| 78 | T3N1 | T2N0M0 | Distal | Cis/5FU + 45 Gy in 25 fr | neoadjuvant | Good | resected |
| 60 | 60mm tumour | T0N0M0 | Distal | Cis/5FU + 45 Gy in 25 Fr | neoadjuvant | *poor* | resected |
| 61 | T3N1 |  | Distal | Cis/5FU + 50.4 Gy in 28 Fr | definitive | Good | non-resected |
| 70 | T3N0 |  | Distal | Cis/5FU + 50.4 Gy in 28 Fr | definitive | Good | non-resected |
| 46 | unknown |  | Distal | Cis/5FU + Rtx. | definitive | *poor* | non-resected |
| 80 | T3 |  | Distal | Cis/5FU + 45 Gy in 25 Fr | definitive | *poor* | non-resected |
| 81 | T3N0 |  | Distal | Cis/5FU + 50.4 in 28 Fr | definitive | *poor* | non-resected |
| 60 | T4 |  | Distal | Cis/5FU + 30 Gy 02 Fr | palliative | Good | non-resected |
| 83 | 30 mm tumour |  | Distal | Cis/5FU + 50.4 Gy in 28 Fr | palliative | Good | non-resected |
| 56 | T4 |  | Distal | Cis/5FU + Rtx. | palliative | *poor* | non-resected |

**5-FU Supplementary Table 2.** miRNA levels in serum small extracellular vesicles from oesophageal cancer patients who responded well vs. poorly to chemoradiotherapy that included 5-FU, for the same miRNAs that were associated with 5-FU response in oesophageal cancer cell lines.

|  | **Good responders (average +/- SE)** | **Poor responders (average +/- SE)** | **Differential Expression** | **Welch's t-test**  **p-value** | **Increased or Decreased in Poor responders** |
| --- | --- | --- | --- | --- | --- |
| **hsa-miR-152-3p** | 0.140 (+/- 0.031) | 0.224 (+/- 0.033) | 0.63 | 0.091 | increased |
| **hsa-miR-31-5p** | 0.003 (+/- 0.002) | 0.033 (+/- 0.014) | 0.09 | 0.099 | increased |
| **hsa-miR-138-5p** | 0.019 (+/- 0.004) | 0.028 (+/- 0.005) | 0.70 | 0.199 | increased |
| **hsa-let-7d-5p** | 0.113 (+/- 0.017) | 0.149 (+/- 0.025) | 0.76 | 0.271 | increased |
| **hsa-miR-126-3p** | 3.354 (+/- 0.413) | 4.262 (+/- 0.655) | 0.79 | 0.279 | increased |
| **hsa-miR-130a-3p** | 0.914 (+/- 0.129) | 1.130 (+/- 0.176) | 0.81 | 0.350 | increased |
| **hsa-miR-660-5p** | 0.529 (+/- 0.097) | 0.349 (+/- 0.153) | 1.51 | 0.352 | *decreased* |
| **hsa-miR-206** | 0.003 (+/- 0.001) | 0.007 (+/- 0.005) | 0.38 | 0.455 | increased |
| **hsa-miR-30d-5p** | 1.182 (+/- 0.257) | 1.028 (+/- 0.129) | 1.15 | 0.603 | *decreased* |
| **hsa-miR-328-3p** | 11.22 (+/- 3.965) | 13.64 (+/- 4.969) | 0.82 | 0.713 | increased |
| **hsa-miR-142-3p** | 2.008 (+/- 0.427) | 2.187 (+/- 0.206) | 0.92 | 0.714 | increased |
| **hsa-miR-203a-3p** | 0.029 (+/- 0.005) | 0.037 (+/- 0.020) | 0.79 | 0.726 | increased |
| **hsa-miR-532-5p** | 0.140 (+/- 0.021) | 0.124 (+/- 0.040) | 1.13 | 0.735 | *decreased* |
| **mmu-miR-93-5p** | 0.503 (+/- 0.04) | 0.478 (+/- 0.148) | 1.05 | 0.880 | *decreased* |
| **hsa-miR-766-3p** | 0.258 (+/- 0.067) | 0.268 (+/- 0.090) | 0.96 | 0.933 | increased |

**5-FU Supplementary Table 3.** House Keeping Genes (HKGs) selected for normalisation of miRNAs isolated from serum small extracellular vesicles of the blood of patients with oesophageal adenocarcinoma who were subsequently treated with a chemoradiotherapy protocol that included 5-FU.

| OpenArray ID | **miRBase v22 sequence** | **miRBase v22 ID or GenBank-ID** | **miRBase or GenBank Accession** |
| --- | --- | --- | --- |
| **000554_hsa-miR-361** | UUAUCAGAAUCUCCAGGGGUAC | hsa-miR-361-5p | MIMAT0000703 |
| **000546_hsa-miR-335** | UCAAGAGCAAUAACGAAAAAUGU | hsa-miR-335-5p | MIMAT0000765 |
| **001097_hsa-miR-146b** | UGAGAACUGAAUUCCAUAGGCUG | hsa-miR-146b-5p | MIMAT0002809 |
| **000402_hsa-miR-24** | UGGCUCAGUUCAGCAGGAACAG | hsa-miR-24-3p | MIMAT0000080 |
| **000545_hsa-miR-331** | GCCCCUGGGCCUAUCCUAGAA | hsa-miR-331-3p | MIMAT0000760 |
| **002098_hsa-miR-223#** | CGUGUAUUUGACAAGCUGAGUU | hsa-miR-223-5p | MIMAT0004570 |
| **000524_hsa-miR-221** | AGCUACAUUGUCUGCUGGGUUUC | hsa-miR-221-3p | MIMAT0000278 |
| **000528_hsa-miR-301** | CAGUGCAAUAGUAUUGUCAAAGC | hsa-miR-301a-3p | MIMAT0000688 |
| **000494_hsa-miR-195** | UAGCAGCACAGAAAUAUUGGC | hsa-miR-195-5p | MIMAT0000461 |
| **000395_hsa-miR-19a** | UGUGCAAAUCUAUGCAAAACUGA | hsa-miR-19a-3p | MIMAT0000073 |
| **000408_hsa-miR-27a** | UUCACAGUGGCUAAGUUCCGC | hsa-miR-27a-3p | MIMAT0000084 |
| **002295_hsa-miR-223** | UGUCAGUUUGUCAAAUACCCCA | hsa-miR-223-3p | MIMAT0000280 |
| **000454_hsa-miR-130a** | CAGUGCAAUGUUAAAAGGGCAU | hsa-miR-130a-3p | MIMAT0000425 |
| **000405_hsa-miR-26a** | UUCAAGUAAUCCAGGAUAGGCU | hsa-miR-26a-5p | MIMAT0000082 |
| **002169_hsa-miR-106a** | AAAAGUGCUUACAGUGCAGGUAG | hsa-miR-106a-5p | MIMAT0000103 |
| **000442_hsa-miR-106b** | UAAAGUGCUGACAGUGCAGAU | hsa-miR-106b-5p | MIMAT0000680 |
| **002281_hsa-miR-193a-5p** | UGGGUCUUUGCGGGCGAGAUGA | hsa-miR-193a-5p | MIMAT0004614 |
| **002283_hsa-let-7d** | AGAGGUAGUAGGUUGCAUAGUU | hsa-let-7d-5p | MIMAT0000065 |
| **002299_hsa-miR-191** | CAACGGAAUCCCAAAAGCAGCUG | hsa-miR-191-5p | MIMAT0000440 |
| **000391_hsa-miR-16** | UAGCAGCACGUAAAUAUUGGCG | hsa-miR-16-5p | MIMAT0000069 |
| **000403_hsa-miR-25** | CAUUGCACUUGUCUCGGUCUGA | hsa-miR-25-3p | MIMAT0000081 |
| **001973_U6-snRNA** | GUGCUCGCUUCGGCAGCACAUAUACUAAAAUUGGAACGAUACAGAGAAGAUUAGCAUGGCCCCUGCGCAAGGAUGACACGCAAAUUCGUGAAGCGUUCCAUAUUUU | U6 snRNA | GenBank: M14486.1 |
